# The complete mitochondrial genome of the servant mouse, *Mus famulus*

**DOI:** 10.1080/23802359.2016.1219627

**Published:** 2016-09-04

**Authors:** Hai-Bo Wu, Yuan Liu

**Affiliations:** a Faculty of Information Engineering and Automation, Kunming University of Science and Technology, Kunming, Yunnan Province, China;; b Faculty of Life Science and Technology, Kunming University of Science and Technology, Kunming, Yunnan Province, China

**Keywords:** The servant mouse, *Mus famulus*, mitochondrial genome

## Abstract

In this article we report the complete mitochondrial genome of the servant mouse, *Mus famulus* for the first time. The genome size is 16,291 bp. The mitogenome contained a total of 13 protein-coding genes, 22 transfer RNA genes, 2 ribosomal RNA genes, and 1 control region. The base composition was A (34.4%), C(24.1%), G (12.2%), and T(29.3%), indicating that the percentage of A and T (63.7%) was higher than that of G and C. Most of these genes were distributed on the H-strand, except for the ND6 subunit gene and eight tRNA genes. A phylogenomic analysis of the 10 complete chloroplast genomes supports that *M. famulus* is a member of the subgenus *Mus*.

Muriod has drawn much attention of researchers over decades, especially *Mus musculus*. With the thorough study on *M. musculus*, it is expected that this animal model provides insight into the workings of other animals and has been widely applied in lab. *Mus famulus* is a poorly known Asiatic species and has been included in the IUCN Red List of threatened species in 2008, only recorded from the Nilgiri Mountains in South India *Mus famulus* was attributed to the subgenus Coelomys by Marshall (1977). Later it was proved that the South Indian species *M. famulus* is a member of the subgenus *Mus* (Chevret & Jenkins 2003). Here, we report the sequence and characteristics of the complete mitogenome of *M. musculus*, which were assembled from high-throughput sequencing data using the Illumina HiSeq platform

(The SRA date in NCBI: ERR019160, ERR019161, ERR019162, ERR019163, ERR019164, ERR019165) and deposited in the GenBank with the accession number KX084803. Annotation of the assembled genome was performed with Dual Organellar GenoMe Annotator (DOGMA) to predict protein coding, transfer RNA (tRNA), and ribosome RNA (rRNA) genes (Wyman et al. 2004). In this study, we included a total of 10 other complete mitochondrial genomes with the obtained cp genome sequence of *M. famulus* to perform a mitochondrial phylogenomic analysis. Maximum-likelihood analyses were implemented in MEGA6 (Tamura et al. 2013). The new data will help to determine the phylogenetic placement of *M. musculus* and fill gaps in our understanding of Mus biology.

The complete mtDNA sequence of *M. famulus* has a total length of 16,291 bp and harboured a total of 13 protein-coding genes, 22 transfer RNA (tRNA) genes, 2 ribosomal RNA (rRNA) genes (12S rRNA and 16S rRNA) and 1 non-coding control region (D-loop). Similar to the other members of Muriod (Hixson et al. 1986; Fernández-Silva et al. 2003), most of the genes of *M. famulus* were located on the H-strand except for the ND6 subunit gene and eight tRNA genes. The base composition was 34.4% of A, 24.1% of C, 12.2% of G, and 29.3% of T, and thus a slight A–T bias (63.7%) was detected. Each of the 13 protein-coding genes was identified and they together encoded 3770 amino acids. The 22 tRNA genes of *M. famulus*, ranging from 59 bp (tRNASer) to 75 bp (tRNALeu), which were determined based on their respective anticodons and secondary structures. Twenty-one of them could be folded into typical clover-leaf secondary structure except for tRNASer in which the dihydrouridine (DHU) arm was lost. The ribosomal subunit genes that encode 12S and 16S rRNA were positioned between the tRNAPhe and tRNALeu genes and further separated by the tRNAVal gene. The OL region was located between tRNAAsn and tRNACys.

Phylogenomic analysis showed that the genus AB042432.1(*M. musculus domesticus*) and NC_005089.1(*M. musculus*), NC_012387.1(*M. musculus castaneus*), AY675564.1(*M. musculus molossinus*) and NC_010339.1(*M. musculus musculus*) form a cluster with strong bootstrap supports, and they together with KX084803(*M. famulus*) form a cluster with strong bootstrap supports (Figure 1). The result supports that *M. famulus* is a member of the subgenus *Mus* (Chevret & Jenkins 2003).

**Figure 1.**
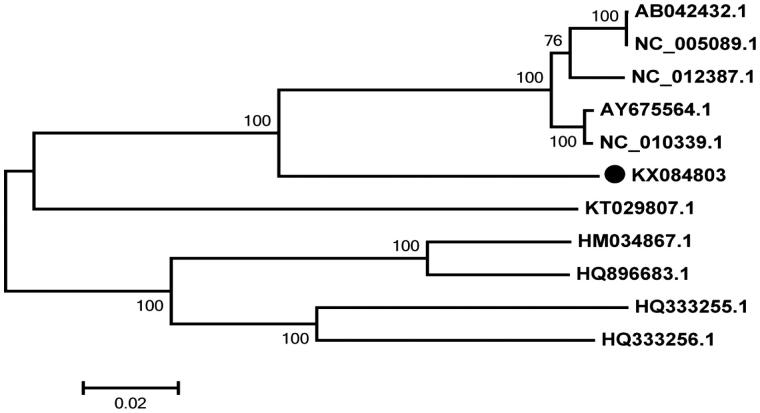
Maximum-likelihood phylogenetic tree based on complete mitochondrial genome sequences of the 10 species. Their accession numbers are as below: AB042432.1, *M. musculus domesticus*; NC_005089.1, *M. musculus*; NC_012387.1, *M. musculus castaneus*; AY675564.1, *M. musculus molossinus*; NC_010339.1, *M. musculus musculus*; KT029807.1, *Bandicota indica*; HM034867.1, *A. chejuensis*; HQ333255.1, *A. draco*; HQ333256.1, *A. latronum*; HQ896683.1, *A. chevrieri*.
